# The Effect of a Nurse‐Led Low‐Carbohydrate Regimen on Symptoms and Eating Triggers of Patients With Metabolic Syndrome: A Quasi‐Experimental Study

**DOI:** 10.1002/hsr2.71934

**Published:** 2026-04-02

**Authors:** Mohammed Faris Abdulghani, Sadeq Al‐Fayyadh, Radhwan Hussein Ibrahim, Osama Ismail Al‐Mashhadany

**Affiliations:** ^1^ College of Nursing Ninevah University Mosul Iraq; ^2^ College of Nursing Baghdad University Baghdad Iraq; ^3^ College of Medicine, Ninevah University Mosul Iraq

**Keywords:** counseling program, eating triggers, metabolic syndrome, nurse‐led, nutrition, quasi‐experimental study, symptoms

## Abstract

**Background and Aim:**

This quasi‐experimental study aimed to determine how a nurse‐led low‐carbohydrate regimen impacts the symptoms and eating triggers experienced by patients with metabolic syndrome.

**Methods:**

This was a quasi‐experimental study. This study recruited 128 participants with metabolic syndrome and assigned them to either an intervention group, which received personalized low‐carb diet support, or a control group, which received standard dietary recommendations. Participants completed questionnaires assessing their symptoms and food triggers before and after the intervention. Data analysis was conducted using SPSS 27.

**Results:**

The prevalence of metabolic syndrome signs was the same in both groups (39.1% vs. 34.4%) before the intervention. After the intervention, a significant reduction in the overall prevalence of these signs and symptoms was observed in the intervention group (12.5%) compared to the control group (43.8%). Furthermore, baseline eating trigger scores showed no significant differences between the groups. Postintervention, the intervention group demonstrated a significant decrease in the total eating trigger score and all subscale scores compared to the control group.

**Conclusion:**

These findings underscore the significance of nurse‐led low‐carbohydrate dietary interventions in influencing symptoms and eating triggers in patients with metabolic syndrome. Applying these insights in clinical practice and research could enhance strategies for effectively managing and preventing metabolic syndrome.

**Trial Registration:** Iranian Clinical Trials Registry (IRCT20231002059587N1).

## 1 Introduction

According to the Adult Treatment Panel III (ATP III) guidelines, metabolic syndrome (MetS) is diagnosed when an individual meets three or more of the following criteria: (1) waist circumference > 102 cm for men and 88 cm for women; (2) serum triglyceride levels of 150 mg/dL or higher; (3) high‐density lipoprotein (HDL) levels below 50 mg/dL for women and below 40 mg/dL for men; (4) fasting blood glucose levels over 100 mg/dL or the use of antidiabetic medications; and (5) elevated blood pressure, with systolic readings of 130 mmHg or more and diastolic readings of 85 mmHg or more [1, 2].

MetS is marked by a combination of risk factors that increase the likelihood of developing atherosclerotic cardiovascular diseases (ASCVD) and type 2 diabetes [3]. These factors contribute to high blood pressure, elevated blood sugar or diabetes, obesity, excess abdominal fat, and abnormal cholesterol or triglyceride levels [4].

The prevalence of MetS has risen sharply over the past 30 years across various sociodemographic groups [5]. MetS is common worldwide, particularly among people with low physical activity and high‐calorie diets [6]. A poor diet increases the likelihood of MetS, while individuals consuming healthier diets often experience a lower risk of obesity and hypertension. Consequently, dietary improvements can play a crucial role in managing MetS [7].

The popularity of low‐carbohydrate diets has grown significantly, particularly for individuals seeking weight loss and disease management strategies. Although the specific composition of these diets varies, they all emphasize reducing carbohydrate intake [8]. These diets are categorized by daily carbohydrate intake: very low carbohydrate, with < 10% carbohydrates or 20–50 g/day; low carbohydrate, with < 26% carbohydrates or under 130 g/day; moderate carbohydrate, containing 26%–44% carbohydrates; and high carbohydrate, with 45% or more of daily caloric intake from carbohydrates [9]. Typically, low‐carb diets reduce carbohydrates while increasing protein and healthy fats [10]. Recently, they have gained attention for managing conditions such as diabetes, obesity, and MetS by improving insulin sensitivity and supporting weight loss [11].

Research has shown that such diets can improve critical indicators of MetS, including blood pressure regulation, blood glucose control, and cholesterol management [12, 13]. Low‐carbohydrate diets can significantly improve waist circumference, blood pressure, cholesterol, triglycerides, and HbA1c levels, potentially lowering the risk of cardiovascular disease and type 2 diabetes in individuals with MetS [14, 15, 16]. A recent RCT showed that a low‐carb diet, compared to a plate‐based diet, could improve glycemic control, promote weight loss, and enhance cardiovascular outcomes [17].

Unhealthy eating patterns and a sedentary lifestyle are key contributors to the development of MetS [7], making dietary changes essential for effective management. Low‐carbohydrate diets, known for their positive effects on weight, blood glucose, lipid levels, and other metabolic markers [15], show promise in addressing these risk factors. However, patient adherence to low‐carbohydrate diets can be challenging due to behavioral and emotional eating triggers. Nurse‐led interventions, incorporating dietary counseling and behavior modification, may provide critical support for sustained adherence and symptom improvement. Research in this area is valuable, as it offers insights into the effectiveness of nurse‐led dietary interventions and may establish a scalable model for managing MetS through targeted nutritional changes and behavioral support. Therefore, this study is vital for developing evidence‐based, nurse‐led dietary protocols to enhance clinical outcomes and nutritional interventions' sustainability for MetS patients. To the researchers' knowledge, previous studies have not examined the efficacy of diets, particularly low‐carbohydrate diets, on symptoms and eating triggers in patients with MetS. Thus, the present study sought to assess the impact of a nurse‐led low‐carbohydrate diet intervention on symptoms and eating triggers in patients with MetS.

## 2 Materials and Methods

### 2.1 Study Type and Setting

This study employed a quasi‐experimental, nonrandomized design with stratified allocation. Participants were stratified by age, gender, and education level to ensure comparability, and then systematically allocated in equal numbers to either the intervention or control group. This study took place at Nineveh University, founded in 1967 and located in Mosul, Iraq. As the second largest and a highly esteemed university in Iraq, following the University of Baghdad, it offers diverse academic programs, including bachelor's, master's, and PhD degrees across fields such as medicine, engineering, humanities, arts, science, nursing, and agriculture. Participants were recruited from two main sources: staff and students at Ninawa University, and patients attending the outpatient clinic of the Research Hospital affiliated with Mosul University. No community residents were included in the sample. This setting provided access to both an academic population and clinical patients, ensuring diversity in participant characteristics.

### 2.2 Sample Size and Sampling

We selected and examined all patients who met specific criteria using purposive sampling. Patients had to meet at least three of the following criteria: abdominal obesity (waist circumference > 102 cm in men or > 88 cm in women), elevated triglycerides (≥ 150 mg/dL (1.7 mmol/L) or on treatment for elevated triglycerides), reduced HDL cholesterol (< 40 mg/dL in men or < 50 mg/dL in women), hypertension (systolic ≥ 130 mmHg and/or diastolic ≥ 85 mmHg, or use of antihypertensive medication), elevated fasting blood sugar (≥ 100 mg/dL or current use of antidiabetic medication). Patients had to be in stable health for 3 months prior to the study, without recent hospitalizations or acute medical issues. Patients with pre‐existing conditions such as cardiac, renal, hepatic diseases, diabetes, pancreatitis, or cancer were ineligible.

We excluded patients who were taking medications for diabetes, hypertension, obesity, or high cholesterol levels. We also excluded those who have participated in other diet or exercise programs in the past 6 months. Eligible patients were then allocated to either the intervention or the control group, ensuring baseline similarity in terms of age, sex, education level, and the severity of their MetS.

We determined the required sample size using an a priori power analysis for independent samples *t*‐test. This method considers the expected effect size, desired power, and significance level. We aimed for a medium effect size (Cohen's *d* = 0.5), a power of 80% (to minimize the chance of type I error), and a significance level of 5% (to control for false positives). Based on these calculations, we included 128 patients in the study. All 128 participants who enrolled completed the intervention and posttest assessments, resulting in a 100% retention rate; therefore, no missing data required imputation or special handling.

### 2.3 Data Collection Tools

We collected data using a comprehensive questionnaire with three main sections.

#### 2.3.1 Demographic and Clinical Information

This section included age, sex, living place, education level, occupation, and lifestyle habits, as well as detailed medical history (previous diagnoses, comorbidities), medication use (current and past treatments relevant to metabolic risk), and family health history (occurrence of diabetes, cardiovascular disease, or metabolic disorders among first‐degree relatives).

#### 2.3.2 Measuring Signs and Symptoms

This researcher‐made tool assessed common manifestations of MetS, including excessive hunger, thirst, frequent urination, tingling sensations, fatigue, frequent infections, necrobiosis lipoidica, acanthosis nigricans, skin changes, headaches, and sleep disturbances. Each item was coded dichotomously (0 = absent, 1 = present). The instrument underwent expert panel review (*n* = 15) for content validity and was pilot‐tested on 15 participants, demonstrating acceptable test–retest reliability (*r* values > 0.80). We administered the questionnaire under controlled conditions to minimize external influences on participant responses.

#### 2.3.3 Food Triggers Questionnaire

This self‐report, researcher‐made tool identified five categories of food consumption triggers: social, emotional, situational, cognitive, and physiological. Responses were rated on a 5‐point Likert scale (1 = *never* to 5 = *always*). Subscale scores were calculated for each category, and a total score was computed by averaging item responses. Validity was established through expert review, and pilot testing confirmed acceptable reliability (Cronbach's *α* = 0.85).

### 2.4 Classification of Triggers

#### 2.4.1 Social Triggers

These triggers arise from social situations that influence eating habits. Examples include increased consumption during social gatherings, altered eating patterns in company versus solitude, and peer pressure impacting food choices.

#### 2.4.2 Emotional Triggers

These triggers are related to emotional states that prompt eating, regardless of physical hunger. Common emotional triggers include eating in response to sadness, stress, anxiety, or even celebrations.

#### 2.4.3 Situational Triggers

These triggers are linked to specific environments or contexts that stimulate eating behaviors. These could include eating while watching TV, in cinemas, during work breaks, or in other non‐food‐focused settings.

#### 2.4.4 Cognitive Triggers

These triggers involve thoughts that lead to eating, even when not hungry. Cognitive distortions about food, such as the belief of finishing one's plate or the perception of certain foods as forbidden, can contribute to unhealthy eating habits.

#### 2.4.5 Physiological Triggers

These triggers are based on physical sensations other than hunger that lead to eating. This might include eating due to perceived low energy levels, mouthwatering smells, or the sight of food, which might be mistaken for hunger cues.

### 2.5 Data Collection

The researcher obtained approval from both the adult nursing department and the Institutional Review Board to conduct the study. The researcher recruited participants from Mosul and obtained informed consent from each participant. Demographic, clinical, food trigger, and symptom data were collected from both the intervention and control groups at the beginning of the study. Participants were stratified based on age, gender, and education level to ensure comparability, and then systematically allocated in equal numbers to either the intervention or the control group.

Participants in the intervention group followed a nurse‐led low‐carbohydrate regimen for 12 weeks. The program consisted of 12 structured educational meetings (1/week), each lasting 30–45 min. The first 4 sessions were delivered in small groups of 5–7 participants, focusing on the principles of a low‐carbohydrate diet, food selection, portion control, and general behavioral strategies. The subsequent sessions were conducted individually, providing tailored dietary counseling, adjustment of meal plans, and personalized strategies for managing food triggers. In addition, participants attended three scheduled one‐on‐one tracking meetings (Weeks 4, 8, and 12) to evaluate adherence, reinforce knowledge, and resolve barriers to dietary compliance. Family members were encouraged to participate in two sessions (the introductory meeting and a mid‐program review) to enhance home‐based support and shared adherence. Knowledge transfer methods included interactive PowerPoint‐based lectures, printed and electronic handouts, practical demonstrations of grocery planning and food preparation, and individual counseling. This combination of group education, personalized meetings, and family engagement was designed to improve both knowledge and sustainability of dietary adherence. Table 1 shows the educational content.

**Table 1:** The educational content of adherence to a low‐carbohydrate diet.

Training	Content
*Patient education and counseling*	
Initial consultation	By explaining the principles of a low‐carbohydrate diet, its potential health benefits, and the challenges associated with adherence, we emphasized the importance of ongoing support and encouragement for patients with metabolic syndrome. This approach helped ensure that patients stay motivated and achieve their health goals.
Diet training	In‐depth information about nutrition, such as calorie intake, macronutrient balance, meal‐timing strategies, supplement considerations, and dietary guidelines. Tailored meal plans to meet specific dietary needs and preferences.
*Individual meal planning*	
Nutritional assessment	Detailed nutritional assessments allowed us to gain a complete picture of each patient's health‐related behaviors, food choices, and nutritional needs.
Identification of food triggers	The Trigger Food Questionnaire assists in identifying the specific social, emotional, situational, cognitive, and physiological triggers for overeating, thereby helping to overcome barriers to participants' success.
Management of food triggers	We taught participants healthy ways to cope with emotional stress, helped them develop strategies for not using unhealthy foods, increased their understanding of situational and cognitive triggers, trained them to focus on the eating experience, recognize hunger and fullness cues, eat slowly to appreciate food, and foster mindful eating practices.
Cooking and shopping tips	To facilitate adherence to a low‐carbohydrate diet, researchers offered practical advice on food preparation techniques, cooking methods, and efficient grocery shopping.

In contrast, the control group continued their existing eating behaviors and received standard care. After 3 months, both groups were reassessed to compare the changes in food triggers and symptoms of MetS. This comparison allowed researchers to evaluate the effectiveness of the intervention.

### 2.6 Data Analysis

Statistical analysis was conducted using IBM‐SPSS 27. Descriptive statistics, including mean ± SD and frequency, were calculated. The *χ* ^2^ test examined MetS symptoms. The *χ* ^2^ test was applied under the assumptions of categorical, independent, and mutually exclusive variables, with expected frequencies ≥ 5 in at least 80% of cells and no expected frequency < 1. The McNemar test was used for paired, dichotomous outcomes (pre‐ and postintervention within the same participants), focusing on discordant pairs. For small discordant counts, the exact binomial version of the McNemar test was considered. The paired *t*‐test was conducted to assess changes within each group, while independent *t*‐test was used to compare differences between the intervention and control groups. The level of significance for all inferential statistical tests in this study was set at *α* = 0.05 (two‐tailed). Results with *p* < 0.05 were considered statistically significant.

## 3 Results

The baseline characteristics of participants revealed no significant differences in mean ages, sex, education level, and living place. Initial systolic blood pressure (SBP) was also comparable between the groups (*p* = 0.793). However, the intervention group exhibited a statistically lower mean DBP (*p* = 0.004). With the exception of smoking habits, where the intervention group reported higher rates and a longer history of smoking (*p* < 0.001), no significant differences were observed in other health indicators and medical histories (*p* > 0.05). Additionally, the intervention group reported consuming fast food more frequently (*p* = 0.007) and cited different reasons for stopping previous diets (*p* = 0.003). Notably, the use of medications for MetS was similar in both groups (*p* > 0.05) (Table 2).

**Table 2:** Comparison of demographic and clinical information of participants between intervention groups and control groups.

Variable	Group					
	Intervention (*n* = 64)		Control (*n* = 64)		Statistical test	*p*
	Mean	SD	Mean	SD		
Age (year)	50.72	6.43	49.14	6.89	*t* = 1.34	0.183
Current SBP (mmHg)	143.83	5.33	144.12	7.31	*t* = −0.26	0.793
Current DBP (mmHg)	90.59	4.29	93.44	6.56	*t* = −2.90	0.004

	*N*	%	*N*	%		
*Age group/year*						
28–33	1	1.6	1	1.6	*χ* ^2^ = 1.43	0.921
34–39	6	9.4	6	9.4		
40–45	7	10.9	11	17.1		
46–51	16	25.0	17	26.6		
52–57	27	42.2	24	37.5		
58–63	7	10.9	5	7.8		
Total	64	100.0	64	100.0		
*Sex*						
Male	32	50.0	31	48.4	*χ* ^2^ = 0.03	0.860
Female	32	50.0	33	51.6		
*Place of residence*						
Rural	3	4.7	5	7.8	*χ* ^2^ = 0.53	0.465
Urban	61	95.3	59	92.2		

	Number	%	Number	%		
*Levels of education*						
Able to read and write	1	1.6	1	1.6	Fisher's exact test = 56	0.336
Primary school	2	3.3	2	3.3		
Secondary school	2	3.3	3	4.7		
Preparatory school	8	12.4	7	10.9		
Undergraduate	26	40.6	20	31.2		
Postgraduate Diploma	7	10.9	5	7.8		
Postgraduate Master	15	23.2	20	31.2		
Postgraduate PhD	3	4.7	6	9.3		
*Occupation*						
Employed	58	90.6	51	79.7	*χ* ^2^ = 3.03	0.082
Unemployed	6	9.4	13	20.3		
*History of HTN*						
Yes	31	48.4	34	53.1	*χ* ^2^ = 0.28	0.596
No	33	51.6	30	46.9		
*History of surgery*						
Yes	11	17.8	20	31.2	*χ* ^2^ = 3.45	0.063
No	53	82.2	44	68.8		
*Obesity*						
Yes	43	67.2	36	56.2	*χ* ^2^ = 1.62	0.203
No	21	32.8	28	43.8		
*Family history of HLD*						
Yes	6	9.4	14	21.9	*χ* ^2^ = 3.79	0.051
No	58	90.6	50	78.1		
*Family history of HD*						
Yes	19	29.7	23	35.9	*χ* ^2^ = 0.57	0.451
No	45	70.3	41	64.1		
*Family history of DM*						
Yes	28	43.8	32	50.0	*χ* ^2^ = 0.50	0.479
No	36	56.2	32	50.0		
*History of smoking*						
Yes	31	48.4	12	18.8	*χ* ^2^ = 12.64	< 0.001
No	33	51.6	52	81.2		
*Smoking duration*						
≤ 5 years	4	6.2	1	1.6	*χ* ^2^ = 12.78	0.002
> 5 years	27	42.2	11	17.2		
No smoking	33	51.6	52	81.2		
*Passive smoking*						
Yes	37	57.8	42	65.6	*χ* ^2^ = 0.83	0.363
No	27	42.2	22	34.4		
*Alcohol consumption*						
Yes	0	0.0	4	6.2	Fisher's exact test = 4.13	0.060
No	64	100.0	60	93.8		
*Number of meals per day*						
1	9	14.0	15	23.4	*χ* ^2^ = 5.03	0.169
2	21	32.8	26	40.6		
3	28	43.8	21	32.8		
4	6	9.4	2	3.2		
*Number of fast‐food meals per week*						
1	9	14.1	9	14.1	*χ* ^2^ = 13.94	0.007
2	15	23.4	20	31.3		
3	6	9.4	13	20.3		
4	2	3.1	8	12.4		
> 4	32	50.0	14	21.9		
*Previous dietary regimens*						
Yes	18	28.1	22	34.4	*χ* ^2^ = 0.58	0.446
No	46	71.9	42	65.6		
*Type of previous diet*						
Low‐carbohydrate	0	0.0	1	1.6	Fisher's exact test = 9.78	0.094
Keto	0	0.0	4	6.3		
Low calories	7	10.9	10	15.6		
Low‐fat	5	7.8	5	7.8		
Metatherian	2	3.1	2	3.1		
Others	4	6.3	0	0.0		
None	46	71.9	42	65.6		
*Reason for stopping diet regimens*						
Difficulty adhering	2	3.1	13	20.3	*χ* ^2^ = 13.12	0.003
Lack of results	8	12.5	6	9.4		
Others	8	12.5	3	4.7		
None	46	71.9	42	65.6		
*Sleep at night/(hours)*						
2	0	0.0	1	1.6	Fisher's exact test = 21.99	< 0.001
3	3	4.7	10	15.6		
4	0	0.0	7	10.9		
5	0	0.0	4	6.3		
6	36	56.3	20	31.3		
7	16	25.0	10	15.6		
8	9	14.0	12	18.7		
*History of using medication to treat metabolic syndrome*						
Yes	2	3.1	6	9.4	*χ* ^2^ = 2.13	0.137
No	62	96.9	58	90.6		

Abbreviations: *χ* ^2^ = chi‐square; DBP = diastolic blood pressure; DM = diabetes mellitus; HD = heart disease; HLD = hyperlipidemia; HTN = hypertension; SBP = systolic blood pressure; SD = standard deviation; *t* = independent *t* test.

Table 3 shows that at baseline, the overall prevalence of total MetS signs and symptoms was similar between groups (39.1% vs. 34.4%, *p* = 0.714). After the intervention, the prevalence significantly decreased in the intervention group (12.5%) compared to the control group (43.8%, *p* < 0.001). For individual symptoms, the intervention group experienced significant reductions in extreme hunger, extreme thirst, frequent urination, tingling in the limbs, shin spots, dry and itchy skin, headaches, and sleep difficulties compared to the control group. In contrast, there were no significant between‐group differences for tiredness, recurrent infections, necrobiosis lipoidica, and acanthosis nigricans.

**Table 3:** Comparison of signs and symptoms of metabolic syndrome in participants between study and control groups, before and after the intervention.

Variable			Group					
			Intervention (*n* = 64)		Control (*n* = 64)		*χ* ^2^ test	*p*
			*N*	%	*N*	%		
Extreme hunger	Before	Yes	12	18.8	20	31.2	2.67	0.152
		No	52	81.2	44	68.8		
	After	Yes	5	7.8	21	32.8	12.36	< 0.001
		No	59	92.2	43	67.2		
McNemar test *p* value			0.092		> 0.999			
Extreme thirst	Before	Yes	11	17.2	11	17.2	0	1
		No	53	82.8	53	82.8		
	After	Yes	3	4.7	12	18.8	6.12	0.013
		No	61	95.3	52	81.2		
McNemar test *p* value			0.008		> 0.999			
Increased or frequent urination	Before	Yes	15	23.4	18	28.1	0.37	0.687
		No	49	76.6	46	71.9		
	After	Yes	6	9.4	19	29.7	8.40	0.007
		No	58	90.6	45	70.3		
McNemar test *p* value			0.012		> 0.999			
Tingling sensations in hands or feet	Before	Yes	14	21.9	9	14.1	1.33	0.357
		No	50	78.1	55	85.9		
	After	Yes	2	3.1	8	12.5	3.90	0.048
		No	62	96.9	56	87.5		
McNemar test *p* value			< 0.001		> 0.999			
Feeling tiredness more than usual	Before	Yes	11	17.2	8	12.5	0.56	0.620
		No	53	82.8	56	87.5		
	After	Yes	6	9.4	6	9.4	0	1
		No	58	90.6	58	90.6		
McNemar test *p* value			0.125		0.500			
Frequent infections (UTI, skin infection, etc.)	Before	Yes	13	20.3	10	15.6	0.48	0.490
		No	51	79.7	54	84.4		
	After	Yes	4	6.3	10	15.6	2.89	0.089
		No	60	93.7	54	84.4		
McNemar test *p* value			0.004		—			
Necrobiosis lipoidica	Before	Yes	12	18.8	6	9.4	2.33	0.127
		No	52	81.2	58	90.6		
	After	Yes	6	9.4	6	9.4	0	1
		No	58	90.6	58	90.6		
McNemar test *p* value			0.031		—			
Acanthosis nigricans (AN)	Before	Yes	14	21.9	8	12.5	1.98	0.160
		No	50	78.1	56	87.5		
	After	Yes	6	9.4	10	15.6	1.14	0.285
		No	58	90.6	54	84.4		
McNemar test *p* value			0.008		0.500			
Shin spots	Before	Yes	9	14.1	8	12.5	0.07	0.795
		No	55	85.9	56	87.5		
	After	Yes	3	4.7	11	17.2	5.13	0.023
		No	61	95.3	53	82.8		
McNemar test *p* value			0.031		0.250			
Dry and itchy skin	Before	Yes	10	15.6	8	12.5	0.26	0.611
		No	54	84.4	56	87.5		
	After	Yes	0	0.0	10	15.6	10.85	0.001
		No	64	100.0	54	84.4		
McNemar test *p* value			< 0.001		0.500			
Headache	Before	Yes	19	29.7	17	26.6	0.16	0.694
		No	45	70.3	47	73.4		
	After	Yes	2	3.1	20	31.3	17.78	< 0.001
		No	62	96.9	44	68.7		
McNemar test *p* value			< 0.001		0.375			
Sleep difficulties	Before	Yes	17	26.6	13	20.3	0.39	0.53
		No	47	73.4	51	79.7		
	After	Yes	3	4.7	16	25	8.90	0.002
		No	61	95.3	48	75		
McNemar test *p* value			< 0.001		0.673			
Sign and symptoms	Before	Yes	25	39.1	22	34.4	0.134	0.714
		No	39	60.9	42	65.6		
	After	Yes	8	12.5	28	43.75	13.95	0.000
		No	56	87.5	36	56.25		
McNemar test *p* value			0.008		> 0.999			

Table 4 illustrates that at the beginning of the study; there were no statistically significant differences in the eating trigger scores between the intervention and control groups (*p* > 0.05). After the intervention, the intervention group exhibited a significant decrease in the total eating trigger score and all subscale scores when compared to the control group (*p* < 0.001). Analysis revealed a significant reduction in the total eating trigger score and all its subscales in the intervention group after the intervention (*p* < 0.05). In the control group, a notable decrease was observed in the total eating trigger score, as well as the social and physiological subscale scores, from before to after the intervention (*p* < 0.05). However, the emotional, situational, and thinking subscale scores did not show significant changes over the study period (*p* > 0.05).

**Table 4:** Comparison of participant eating triggers between intervention and control groups, before and after the intervention.

Variable		Group					
		Intervention (*n* = 64)		Control (*n* = 64)		Independent *t*‐test	*p*
		Mean	SD	Mean	SD		
Social	Before	4.05	2.06	3.69	1.82	1.06	0.293
	After	1.85	0.55	3.36	1.55	−7.37	< 0.001
Paired *t*‐test		−9.75		−3.15			
*p*		< 0.001		0.002			
Emotional	Before	3.78	2.83	3.78	2.28	< 0.001	> 0.999
	After	2.02	0.72	3.71	2.24	−5.72	< 0.001
Paired *t*‐test		−7.38		−1.98			
*p*		< 0.001		0.051			
Situational	Before	3.43	2.25	3.39	2.17	0.11	0.913
	After	1.70	0.51	3.39	2.17	−6.06	< 0.001
Paired *t*‐test		−6.73		—			
*p*		< 0.001		—			
Thinking	Before	3.16	1.56	3.13	1.57	0.10	0.922
	After	2.28	0.83	3.07	1.44	−3.79	< 0.001
Paired *t*‐test		−5.44		−1.669			
*p*		< 0.001		0.100			
Physiological	Before	5.70	2.90	5.55	2.82	0.31	0.758
	After	2.14	0.91	5.23	2.29	−9.01	< 0.001
Paired *t*‐test		−10.99		−2.80			
*p*		< 0.001		0.007			
Paired *t*‐test	Before	4.02	1.08	3.91	1.06	0.62	0.534
	After	2.0	0.34	3.75	0.98	−13.47	< 0.001
Paired *t*‐test		−17.21		−4.65			
*p*		< 0.001		< 0.001			

## 4 Discussion

This study sought to determine the effect of a nurse‐led low‐carbohydrate dietary intervention on the symptoms and eating triggers in patients with MetS. The results indicate that the nurse‐led low‐carbohydrate intervention produced meaningful improvements in several key symptoms of MetS. Specifically, extreme hunger, extreme thirst, frequent urination, paresthesia in the limbs, shin spots, dry and itchy skin, headaches, sleep problems, and the overall symptom burden were significantly reduced compared with the control group. However, symptoms such as tiredness, frequent infections, necrobiosis lipoidica, and acanthosis nigricans did not show significant differences between groups, which suggests that some manifestations of MetS may be less responsive to short‐term dietary change.

The results obtained in this study can be attributed to several potential explanations. One potential contributing factor is the low‐carbohydrate diet followed by the intervention group, which typically involves reducing carbohydrate intake, particularly refined sugars and processed foods [18]. It is well‐documented that a low‐carbohydrate diet can positively affect blood sugar control and insulin sensitivity [11]. By reducing carbohydrate intake, the intervention group may have experienced improved regulation of blood glucose levels, which could have decreased symptoms such as extreme hunger, extreme thirst, and frequent urination.

Additionally, low‐carbohydrate diets have been associated with weight loss and improved body composition, including reductions in body fat and abdominal obesity [19]. It is reasonable to suggest that the intervention group experienced weight loss or improvements in body composition, which decreased symptoms such as paresthesia in the extremities, leg spots, and dry and itchy skin.

Furthermore, low‐carbohydrate diets can impact various hormonal and metabolic pathways. They can lower insulin levels, increase ketone production, and modify lipid profiles [20]. Low‐carbohydrate diets may help regulate appetite and alleviate feelings of extreme hunger by reducing insulin levels. Changes in lipid profiles can positively affect cardiovascular health [11] and potentially reduce symptoms such as headaches.

Moreover, specific dietary patterns, including low‐carbohydrate diets, possess anti‐inflammatory effects [21]. These diets may reduce inflammation in the body, which could contribute to the observed decrease in symptoms such as sleep problems and necrobiosis lipoidica in the intervention group.

It is also possible that part of the symptom reduction observed in the intervention group was influenced not only by actual physiological improvement, but also by increased health knowledge, awareness of bodily cues, and enhanced self‐regulation following the educational sessions. The nurse‐led counseling may have heightened participants' recognition of metabolic risk factors and improved their self‐monitoring of eating patterns and symptoms. Thus, changes in self‐reported symptom frequency may reflect both behavioral modification and enhanced symptom interpretation rather than purely biological improvement.

Based on the available research, previous studies have examined the effects of low‐carbohydrate diets on MetS symptoms. However, none have specifically focused on hunger, thirst, and frequency of urination. To compare the results of this particular aspect of the present study, the researchers referred to similar studies that examined the role of low‐carbohydrate diets in managing MetS.

For instance, a study conducted by Mirmiran and colleagues revealed that low‐carbohydrate diets high in protein and fat may be associated with a reduced risk of MetS [22]. Conversely, it has been reported that a high‐carbohydrate, low‐fat diet can potentially worsen MetS [23]. Furthermore, a meta‐analysis of randomized controlled clinical trials indicated that low‐carbohydrate diets are at least as effective as low‐fat diets in losing weight and improving metabolic risk factors [24]. These findings support the present study's results, emphasizing the significance of low‐carbohydrate diets in managing MetS.

However, not all studies have found an association between a low‐carbohydrate diet and MetS. For instance, the survey conducted by Shirani and colleagues did not identify any significant correlation between this diet and MetS [25]. Similarly, a study by Eslamian and colleagues also failed to find a significant association between a low‐carbohydrate diet and the risk of MetS [26].

The lack of consistent findings may be attributed to several factors. First, the duration of the dietary intervention can influence the results, as short‐term studies may not fully capture the effects. Second, compliance and adherence to a low‐carbohydrate diet can vary among participants, leading to variability in the study results. Additionally, variations in the macronutrient composition of low‐carbohydrate diets, such as the specific fat, protein, and carbohydrates ratio, could contribute to the conflicting findings. Other influential factors include the baseline health status of the participants, methodological limitations, and differences in study design, including sample size and control of confounding variables.

The results of the present study revealed a significant decrease in the total score of food stimuli and its subscales in the intervention group following the low‐carbohydrate diet intervention compared to the control group.

These findings can be attributed to several factors. First, a low‐carbohydrate diet helps stabilize blood sugar levels and insulin response by restricting the intake of refined sugars and starches, which in turn reduces cravings [27]. Second, the increased satiety resulting from consuming higher amounts of protein and healthy fats in a low‐carb diet reduces overall appetite. Protein and healthy fats promote feelings of fullness and can contribute to decreased cravings and snacking between meals. Additionally, hormonal changes associated with carbohydrate restriction, such as increased levels of PYY and GLP‐1, may contribute to appetite suppression [28]. The state of ketosis, which can occur with deficient carbohydrate intake, may also play a role due to the appetite‐suppressing properties of ketones [29].

However, the reductions in food triggers observed in this study cannot be attributed to dietary modification alone. The intervention also incorporated behavior therapy and trigger management, which included identifying personal food triggers and providing strategies for coping with social, emotional, situational, cognitive, and physiological cues. This component is particularly important because this shows that while the intervention group demonstrated significant reductions across all trigger domains, the control group improved only in the social and physiological subscales. Several factors may account for these limited yet notable changes. First, merely participating in the study and completing the Food Trigger Questionnaire may have increased participants' awareness of their eating habits, particularly those driven by environmental cues (social situations) and bodily sensations (physiological hunger or cravings). This heightened awareness—consistent with the Hawthorne effect—can independently influence behavior even in the absence of a formal intervention [30]. Second, social and physiological triggers tend to be more sensitive to short‐term, naturally occurring fluctuations, such as changes in daily routines, meal timing, or seasonal variation in appetite [31, 32]. Finally, participants in the control group may have unintentionally adopted minor dietary adjustments as a result of reflecting on their behavior during data collection, which could reduce externally prompted or physiologically driven eating episodes. These contextual improvements contrast sharply with the more complex emotional, situational, and cognitive triggers, which showed no significant change in the control group and are typically resistant to modification without structured behavioral counseling. This distinction reinforces the importance of the nurse‐led behavioral component in producing comprehensive and sustained reductions in maladaptive eating triggers.

Taken together, the improvements are best explained as a synergistic effect of dietary control and behavioral trigger management. This dual approach is consistent with evidence from prior studies using tools such as the Three Factor Eating Questionnaire [33], the Food Craving Questionnaire [34], and other qualitative or case‐based reports [35, 36, 37, 38, 39, 40], which also confirm that ketogenic or low‐carbohydrate diets are linked to reduced hunger and cravings. Nonetheless, the present study adds to this body of evidence by demonstrating that when combined with structured behavioral therapy, the reductions extend beyond physiological drivers to encompass complex emotional and cognitive triggers. Further research using designs that separate dietary and behavioral effects will be essential to validate and generalize these findings.

### 4.1 Limitations

While this well‐designed, multiphase study had strong methods, some limitations should be noted. One limitation of this study is that the absence of community residents may reduce the generalizability of the findings, as individuals affiliated with educational institutions could have higher baseline health literacy and greater readiness to adopt dietary changes compared to the general population. The small number of participants limits how widely the intervention's results can be applied. Additionally, the 3‐month intervention period might not be long enough to see the lasting effects and consistency of dietary changes in patients with MetS. The study also depended on participants to accurately report their diet, which could introduce some errors. Another limitation relates to the measurement of outcomes. Signs and symptoms of MetS were self‐reported, which may introduce reporting bias due to increased awareness following the educational intervention. Future studies should include objective physiological biomarkers such as HDL levels, triglycerides, fasting glucose, blood pressure, and waist circumference, in addition to self‐report measures, to provide a more comprehensive assessment of intervention effectiveness. Finally, there are not many national publications supporting these findings.

## 5 Conclusion

The study indicated that nurse‐led low‐carbohydrate dietary interventions can lead to notable improvements in symptoms and eating triggers in patients with MetS. Nurses are essential in promoting metabolic health by training and assisting patients in following these diets through personalized counseling, meal plans, behavioral support, follow‐up, collaboration, and empowerment. Incorporating these results into healthcare and future studies could enhance approaches for the successful management and prevention of MetS. Although these findings are promising, further research is needed to determine long‐term outcomes, optimal carbohydrate levels, and underlying mechanisms.

## Author Contributions

All authors contributed to data collection, data analysis, and writing of the article.

## Funding

The authors received no specific funding for this work.

## Disclosure

The lead author Mohammed Faris Abdulghani affirms that this manuscript is an honest, accurate, and transparent account of the study being reported; that no important aspects of the study have been omitted; and that any discrepancies from the study as planned (and, if relevant, registered) have been explained.

## Ethics Statement

This research was conducted after obtaining ethical approval from Baghdad (Ref: 1540) on May 8, 2023, and was registered with the Iranian Clinical Trials Registry (IRCT20231002059587N1). The study adhered to the Consolidated Standards of Reporting Trials (CONSORT) guidelines.

## Consent

Participants were informed about the study's objectives and the potential benefits of their participation. They provided written informed consent and were assured of confidentiality. It was emphasized that their decision to participate would not affect their treatment.

## Conflicts of Interest

The authors declare no conflicts of interest.

## Data Availability

Data are available on request from the authors.
